# Transcriptome analysis reveals potential function of long non-coding RNAs in 20-hydroxyecdysone regulated autophagy in *Bombyx mori*

**DOI:** 10.1186/s12864-021-07692-1

**Published:** 2021-05-22

**Authors:** Huili Qiao, Jingya Wang, Yuanzhuo Wang, Juanjuan Yang, Bofan Wei, Miaomiao Li, Bo Wang, Xiaozhe Li, Yang Cao, Ling Tian, Dandan Li, Lunguang Yao, Yunchao Kan

**Affiliations:** 1 China-UK-NYNU-RRes Joint Laboratory of insect biology, Henan Key Laboratory of Insect Biology in Funiu Mountain, Nanyang Normal University, 473061 Nanyang, Henan, China; 2 School of Life Science, Zhengzhou University, 450001 Zhengzhou, Henan, China; 3 State Key Laboratory of Silkworm Genome Biology / Biological Science Research Center, Southwest University, 400716 Chongqing, China; 4 Guangdong Laboratory for Lingnan Modern Agriculture/Guangdong Provincial Key Laboratory of Agro-animal Genomics and Molecular Breeding, College of Animal Science, South China Agricultural University, 510642 Guangzhou, Guangdong China

**Keywords:** LncRNA, Transcriptome, 20-hydroxyecdysone, Autophagy-related gene, Silkworm

## Abstract

**Background:**

20-hydroxyecdysone (20E) plays important roles in insect molting and metamorphosis. 20E-induced autophagy has been detected during the larval–pupal transition in different insects. In *Bombyx mori*, autophagy is induced by 20E in the larval fat body. Long non-coding RNAs (lncRNAs) function in various biological processes in many organisms, including insects. Many lncRNAs have been reported to be potential for autophagy occurrence in mammals, but it has not been investigated in insects.

**Results:**

RNA libraries from the fat body of *B. mori* dissected at 2 and 6 h post-injection with 20E were constructed and sequenced, and comprehensive analysis of lncRNAs and mRNAs was performed. A total of 1035 lncRNAs were identified, including 905 lincRNAs and 130 antisense lncRNAs. Compared with mRNAs, lncRNAs had longer transcript length and fewer exons. 132 lncRNAs were found differentially expressed at 2 h post injection, compared with 64 lncRNAs at 6 h post injection. Thirty differentially expressed lncRNAs were common at 2 and 6 h post-injection, and were hypothesized to be associated with the 20E response. Target gene analysis predicted 6493 lncRNA-mRNA *cis* pairs and 42,797 lncRNA-mRNA *trans* pairs. The expression profiles of *LNC_000560* were highly consistent with its potential target genes, *Atg4B*, and RNAi of *LNC_000560* significantly decreased the expression of *LNC_000560 and Atg4B*. These results indicated that *LNC_000560* was potentially involved in the 20E-induced autophagy of the fat body by regulating *Atg4B*.

**Conclusions:**

This study provides the genome-wide identification and functional characterization of lncRNAs associated with 20E-induced autophagy in the fat body of *B. mori*. *LNC_000560* and its potential target gene were identified to be related to 20-regulated autophagy in *B. mori*. These results will be helpful for further studying the regulatory mechanisms of lncRNAs in autophagy and other biological processes in this insect model.

**Supplementary Information:**

The online version contains supplementary material available at 10.1186/s12864-021-07692-1.

## Background

Macroautophagy (hereafter autophagy) is an essential, evolutionarily conserved cellular degradation and recycling process in all eukaryotes [1]. The role of autophagy is to maintain cellular homeostasis by degrading intracellular components. Autophagy is a process involving induction, cargo recognition and packaging, vesicle formation, and breakdown. A series of autophagy-related (*Atg*) genes are required for the initiation, nucleation, expansion, and completion of bodies known as autophagosomes, which eventually fuse with lysosomes [2]. Autophagy is essential to many physiological and developmental processes, and defects in autophagy are often associated with diseases and tumor progression [3, 4].

Autophagy is regulated by several ATG proteins, which are evolutionary conserved from yeast to mammals, ATG proteins are classified into six functional complexes including ATG1-kinase complex, phosphatidylinositol-3-kinase complex, ATG2-ATG18 complex, ATG9 membrane protein, ATG8 conjugation system and ATG12 conjugation system [5]. ATG4 is the only cysteine protease specific to ATG8, and essential for the conjugation and deconjugation of ATG8. Although ATG4 and ATG8 are evolutionarily conserved, higher eukaryotes have multiple homologs for both proteins. In contrast to the Atg4 and Atg8 in yeast, there are four ATG4 and six ATG8 homologs in mammals, the protease activity of the ATG4 homologs is markedly different, but ATG4B exhibits much higher activity than the other homologs [6, 7]. ATG4 homologs are important for autophagosome formation, autophagy is inhibited by suppressing ATG4 expression [8]. In *B. mori*, 15 Atgs have been identified in the genome [9, 10], and include two Atg4 homologs, Atg4B and Atg4-like, but their function are still not characterized.

In insects, autophagy is an important physiological process during metamorphosis. The molting and metamorphosis of insects are regulated primarily by 20-hydroxyecdysone (20E) and juvenile hormone (JH) [11, 12]. 20E-induced autophagy can be detected during the larval–pupal transition in different insects as well as in *B. mori*. In the fat body, the ecdysone receptor (EcR) is necessary for the induction of autophagy by 20E in *Drosophila melanogaster* and *B. mori*, while *Atg* genes are upregulated in *B. mori* during molting and pupation [10, 13, 14].

Long non-coding RNAs (lncRNAs) are a large class of RNA transcripts that are longer than 200 nucleotides and lack protein-coding potential [15]. The majority of lncRNAs are transcribed by RNA polymerase II, and processed by 5’-capping, 3’-polyadenylation, and alternative splicing, similar to mRNAs [16]. In the last decades, advances in transcriptome sequencing have led to the identification of a large number of lncRNAs in various eukaryotic organisms using new technologies and bioinformatics methods [17–22], but there are still few studies into their functions. Recently, lncRNAs have attracted attention because of their critical roles in organismal growth, development, senescence, and death. There is evidence that lncRNAs participate in a range of biological processes, such as X-chromosome silencing [23], dosage compensation [24], chromosome modification [25], genomic imprinting [26], and control of gene expression [27].

Several studies have investigated the regulatory mechanisms of lncRNAs in autophagy from multiple aspects in mammals. For example, high glucose levels have been shown to reduce the expression of the lncRNA *H19*, which activates the transcription of DIRAS3 and induces autophagy by repressing the PI3K/AKT/mTOR pathway [28]. LncRNAs *HOTAIRM* promote the initiation of autophagy by increasing ULK expression [29]. The lncRNAs *PCGEM1* affect the nucleation of autophagy by regulating the expression of *Beclin1* [30]. The lncRNAs *APF* can promote the extension and fusion of autophagosomes [31]. The lncRNA *MALAT1* can also regulate the expression of *LAMP1* by miR-23-3p during the formation of autolysosomes [32]. However, research into insect lncRNAs and their functions during metamorphosis and autophagy is still scarce.

The silkworm *B. mori*, an economically important insect which undergoes complete metamorphosis, is a good model to study the role of lncRNAs in autophagy. The fat body is an organ which is important for nutrient storage and energy metabolism in insects. It plays critical roles in the regulation of insect metamorphosis by coordinating different hormones and nutritional signals [33, 34]. Previous studies have shown that the injection of 20E into actively feeding larvae up-regulates *Atg* genes and reduces TORC1 activity, inducing autophagy in the fat body [10]. Although some progress has recently been made on the investigation of silkworm lncRNAs [35–41], their functions remain poorly understood, including whether and how they regulate autophagy and metamorphosis. In this study, the lncRNAs associated with the 20E response in the fat body of *B. mori* were identified and validated. The function of the selected lncRNA was further characterized by studying the specific expression patterns and its target gene. To the best of our knowledge, our study was the first to identify lncRNA that might be involved in the 20E-induced autophagy in silkworm. Our results lay a foundation for future studies in elucidating the regulatory role of lncRNAs in autophagy and other biological processes in *B. mori*.

## Results

### Genome-wide identification of lncRNAs using RNA-seq

**Fig. 1:**
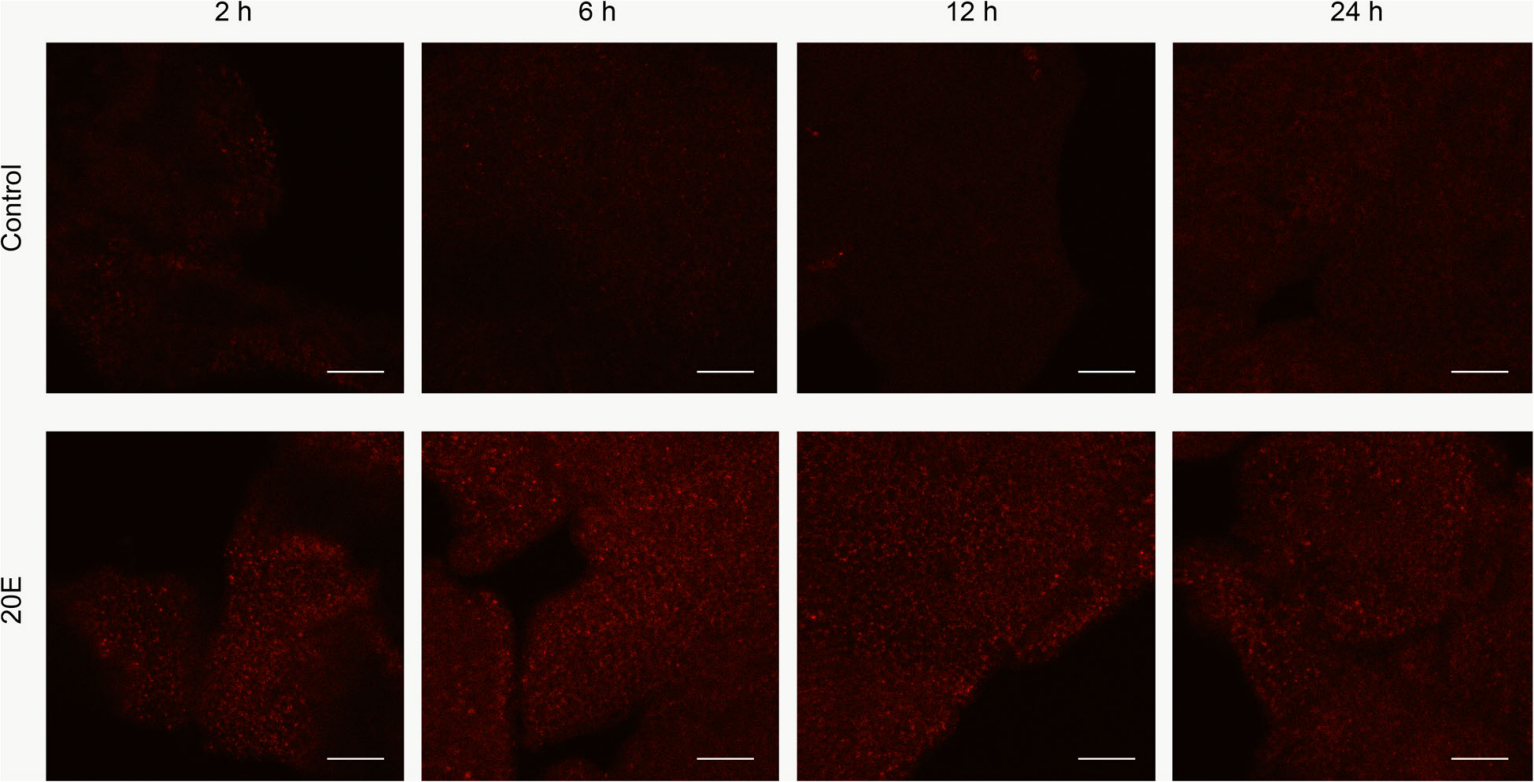
Autophagy detection after 20E treatments in *B. mori* fat body by LysoTracker Red staining (red, magnification 40 x, the scale is 50 μm)

**Fig. 2:**
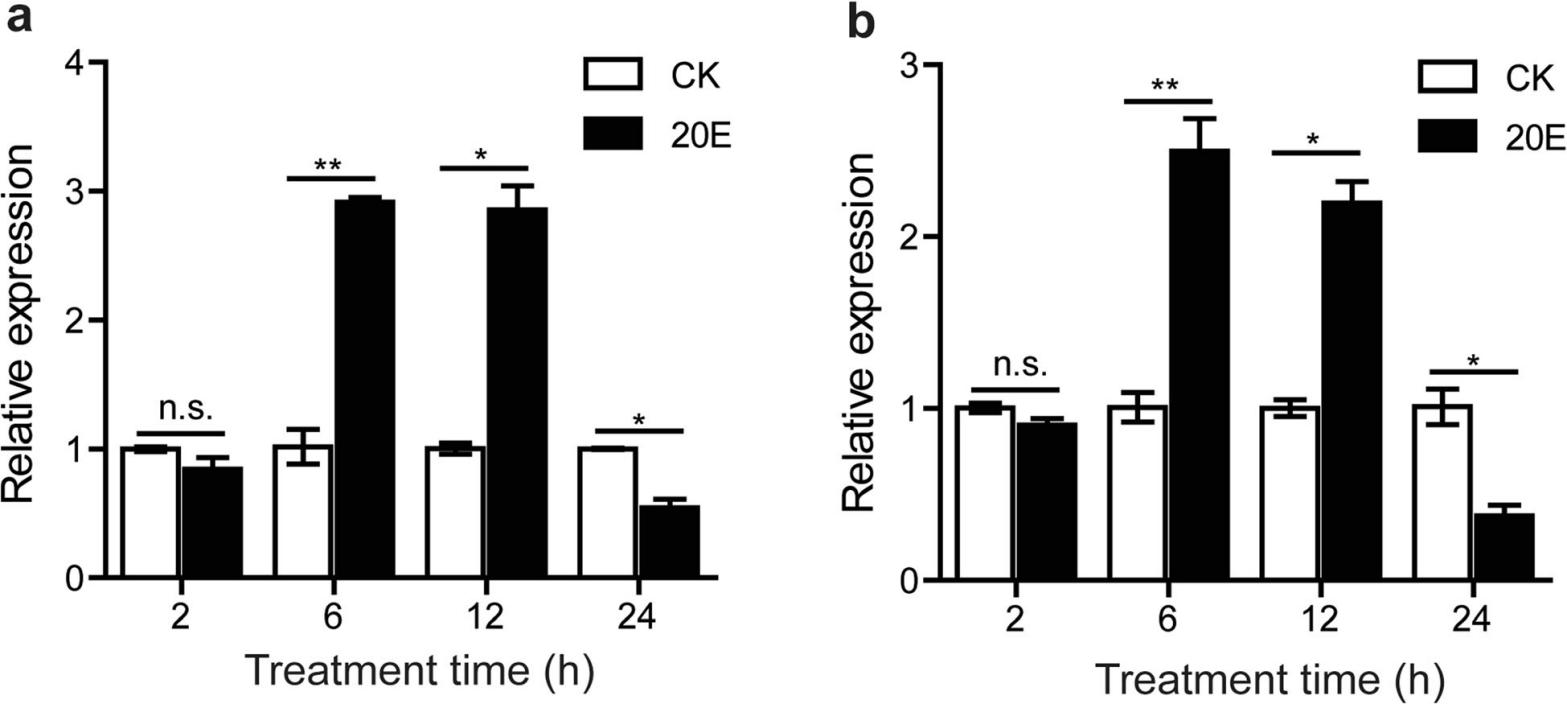
Expression analysis of *Atg1* (**a**) and *Atg8* (**b**) in 20E treated fat body by qRT-PCR. Data were normalized to the housekeeping gene *actinA3* and are shown as the mean ± standard error, *P < 0.05, **P < 0.01, no significant differences are denoted by n.s. above bars, Two tailed, paired t test

We first estimated the 20E-induced autophagy in the fat body from the 2-day-old 5th instar larvae using LysoTracker Red staining. The staining was undetectable in the control samples. However, in the 20E-induced samples, the staining increased in fat body 2–12 h post-injection with 20E (h.p.i.20E) but became less intense at 24 h.p.i.20E (Fig. 1). The expression of the autophagy-related genes, *Atg1* and *Atg8*, in the fat body at 2, 6, 12, and 24 h.p.i.20E showed that their expression did not change at 2 h.p.i.20E, but had increased significantly at 6 and 12 h.p.i.20E, then decreased at 24 h.p.i.20E (Fig. 2).

Based on these results, and those of previous studies [10, 42], 12 libraries from controls and treated fat bodies at 2 and 6 h.p.i.20E were constructed and sequenced. Approximately 82 to 108 million raw reads were generated per sample, and 80 to 105 million clean reads per sample with high quality were retained. Approximately 85.12–90.61 % of clean reads were mapped to the silkworm genome, and 75.91–85.27 % of clean reads were uniquely mapped. The clean reads were mapped with a reference annotation, and 56.67–64.67 % of them were mapped to mRNAs (Additional file 1: Table S1).

**Fig. 3:**
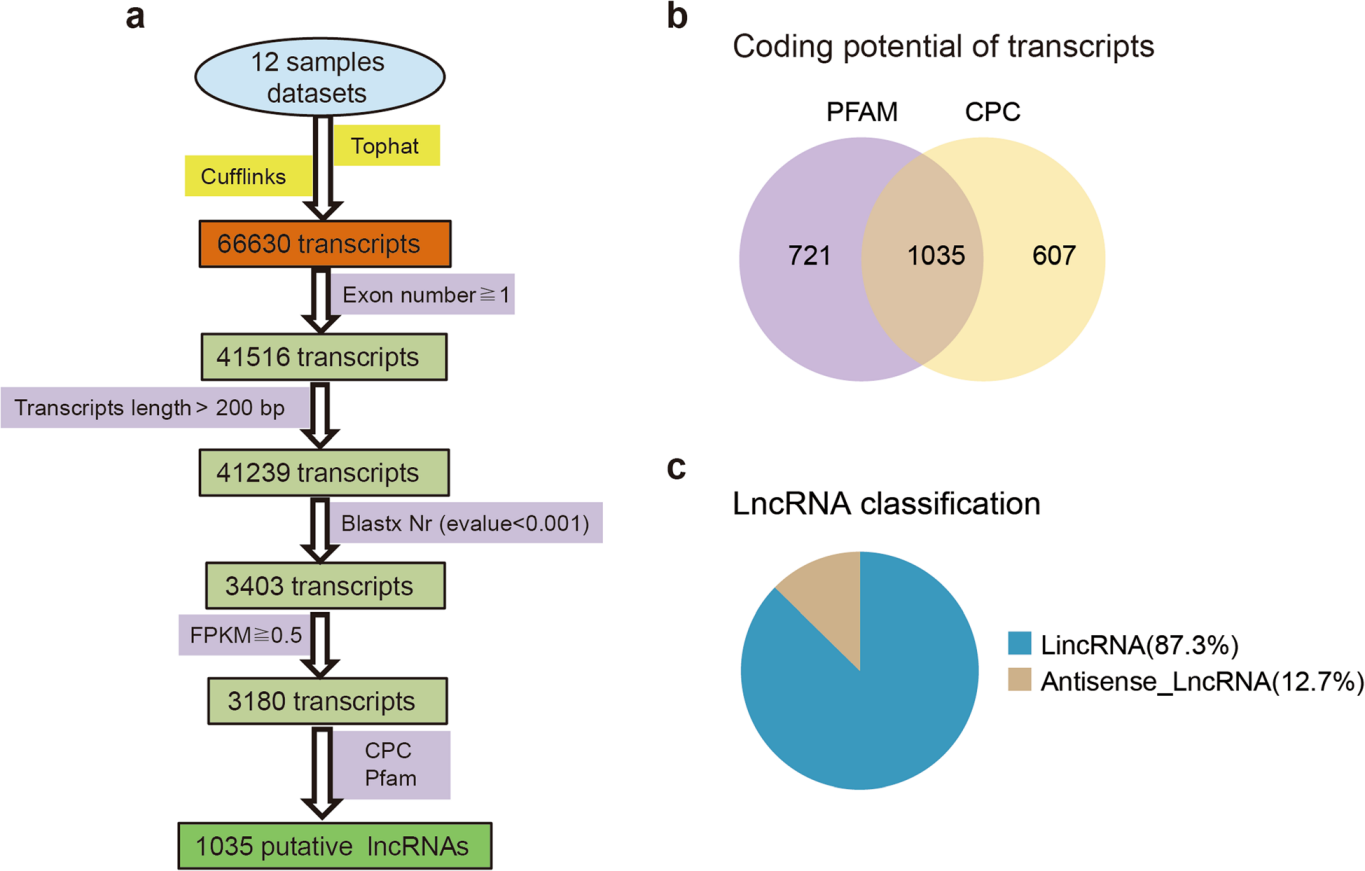
The computational pipeline for identifying lncRNAs from RNA-seq data of silkworm fat body and their classification. **a** The filter pipeline for identification of lncRNAs. **b** Identification of lncRNAs using PFAM and CPC. **c** The classification of identified lncRNAs

The putative lncRNAs were identified following several filtering steps (Fig. 3a). The protein-coding potential of each transcript was predicted using the Coding Potential Calculator (CPC, http://cpc.gao-lab.org/) and the Protein Families Database (PFAM) [43] (Fig. 3b). In total, 1035 putative lncRNAs were identified. According to their genomic location and neighboring genes, they were classified into two types: lincRNA (long intergenic non-coding RNA) and antisense lncRNA. LincRNAs are transcripts located in the intergenic regions between two protein-coding genes. Antisense lncRNAs are transcripts that have exonic overlap with a known protein-coding gene on the opposite strand. A total of 87.3 % of the identified lncRNAs were lincRNAs, and 12.7 % were antisense lncRNAs (Fig. 3c). Information about all lncRNAs is shown in Additional file 2 (Table S2).

### Characteristic features of lncRNAs and mRNAs

**Fig. 4:**
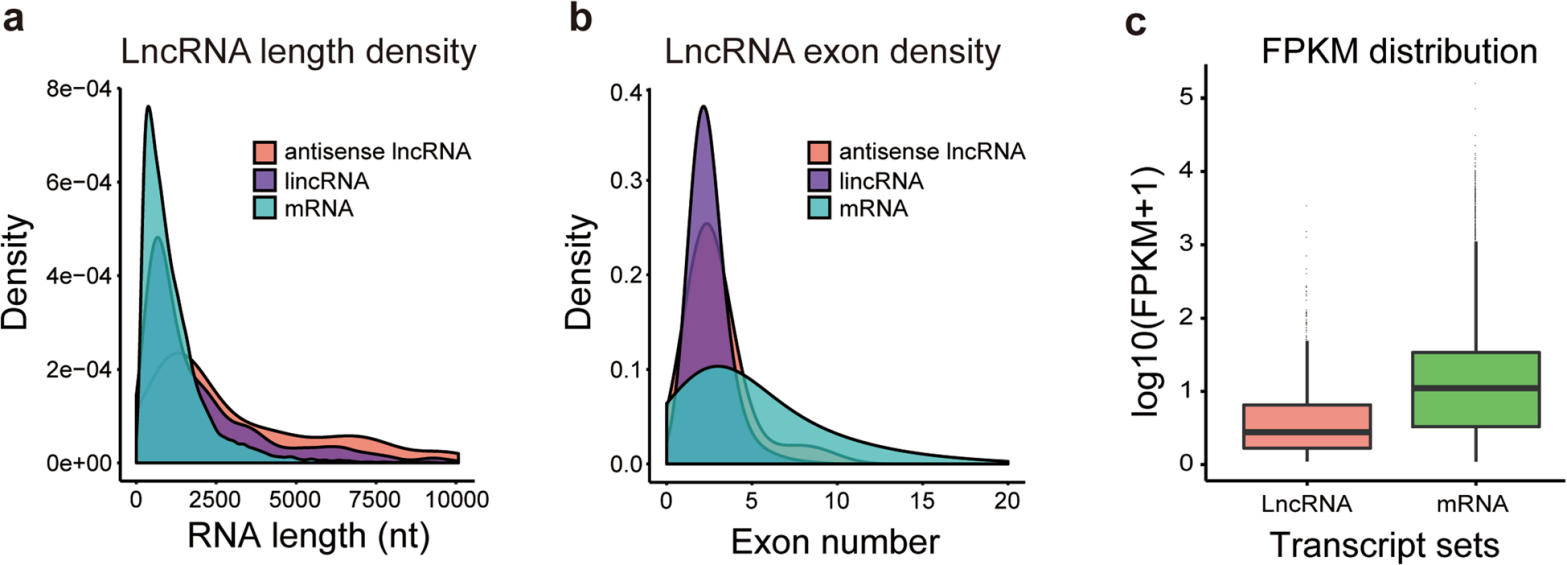
Features of silkworm lncRNAs and mRNAs. **a** Transcript size distribution of lincRNAs, antisense lncRNAs, and mRNAs. **b** Number of exons per transcript of lincRNAs, antisense lncRNAs, and mRNAs. **c** Expression level indicated by log10(FPKM + 1) in the lncRNAs and mRNAs

A total of 1035 lncRNAs and 14,622 mRNAs were obtained from the fat body of silkworm (Additional file 3: Table S3). The features of the lncRNAs, including transcript length, exon number, and expression levels, were assessed and compared with those of mRNAs. The size of the lncRNAs varied from 212 nt to 42,442 nt, with 60 % of lncRNAs having a length ≥ 1000 nt. The mean length of lincRNAs was 2412 nt, and that of antisense lncRNAs was 4027 nt, greater than the mean length of mRNAs (1224 nt) (Fig. 4a). LncRNAs had fewer exons than mRNAs: 2.48 for lincRNAs and 2.95 for antisense lncRNAs versus 5.44 for mRNAs on average (Fig. 4b), and the expression level of lncRNAs was lower than that of mRNAs (Fig. 4c). These results provided an overview of transcriptional changes in the expression of lncRNAs in the silkworm fat body in response to 20E treatment.

### Differential expression of lncRNAs and mRNAs

**Fig. 5:**
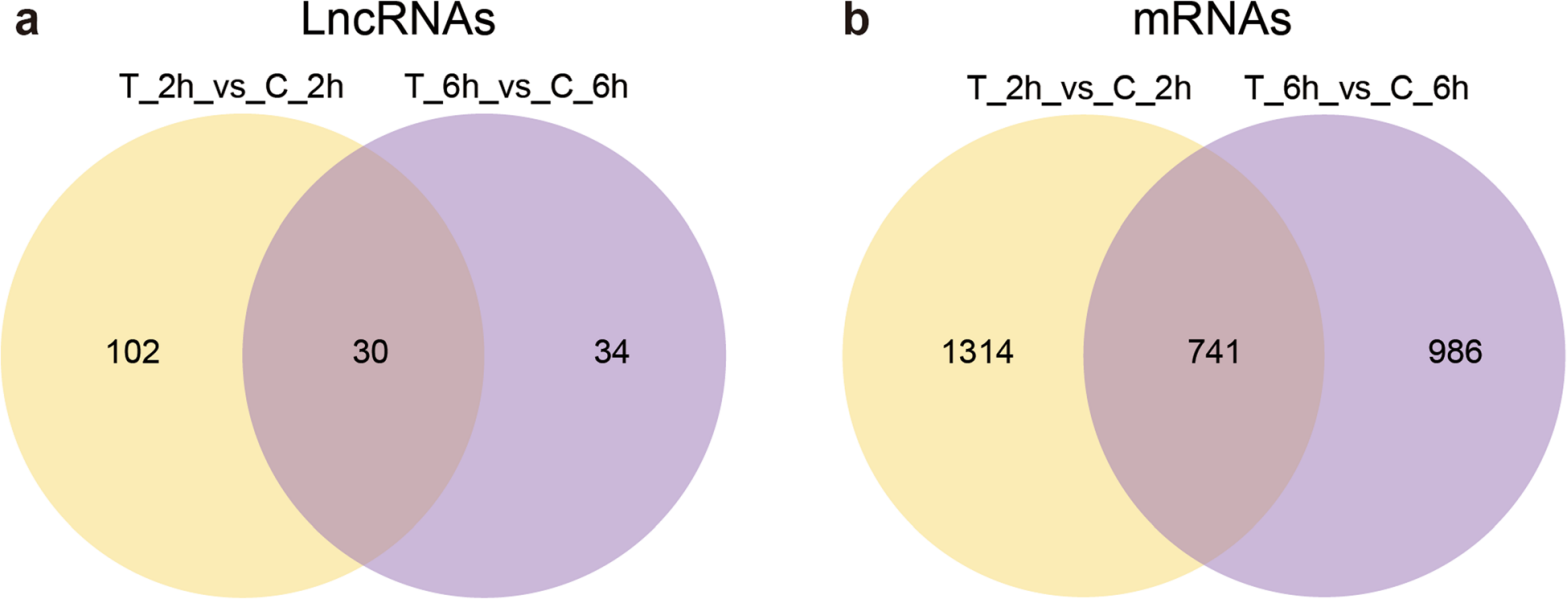
Overlapped differentially expressed lncRNAs (**a**) and mRNAs (**b**) in T_2h vs. C_2h and T_6h vs. C_6h

**Fig. 6:**
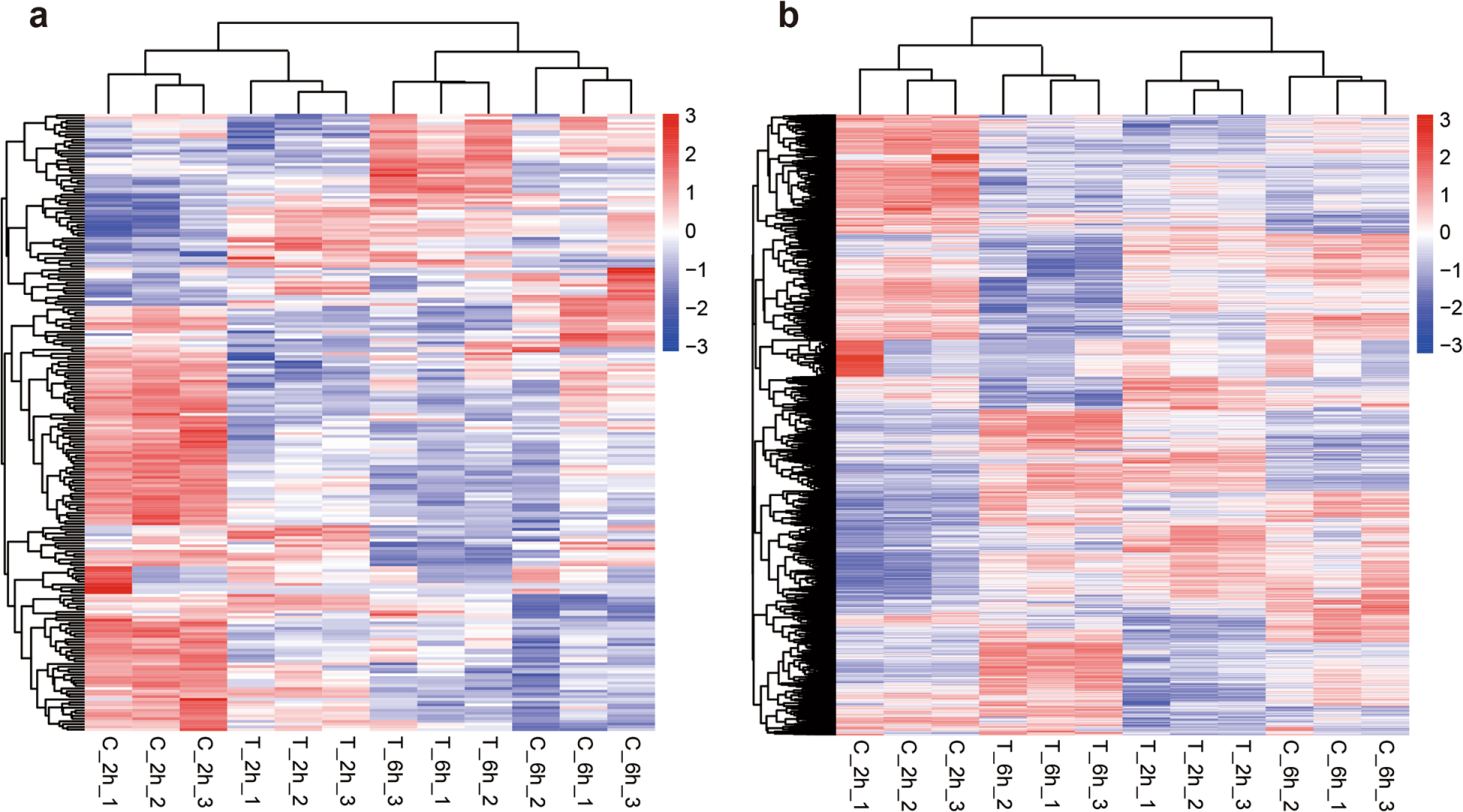
Hierarchical clustering of the differentially expressed lncRNAs (**a**) and mRNAs (**b**) in T_2h vs. C_2h and T_6h vs. C_6h

Expression changes of lncRNAs and mRNAs in different silkworm samples were investigated based on the Fragments Per Kilobase of exon model per Million mapped fragments (FPKM) values of genes. In total, 166 differentially expressed lncRNAs and 3041 mRNAs were detected in silkworm fat body after 20E injection (Fig. 5). Thirty-five upregulated and 97 downregulated lncRNAs were found at 2 h.p.i.20E between treated and control (T_2h vs. C_2h), 31 upregulated and 33 downregulated lncRNAs were found at 6 h.p.i.20E between treated and control (T_6h vs. C_6h), 878 upregulated and 1177 downregulated mRNAs were detected in the T_2h vs. C_2h group, and 755 upregulated and 972 downregulated mRNAs in the T_6h vs. C_6h group (Table 1). As shown in Figs. 5 and 30 differentially expressed lncRNAs and 741 differentially expressed mRNAs were shared between the two groups (Additional file 4: Table S4). Heatmaps constructed from these data are shown in Fig. 6.

**Table 1:** Number of differentially expressed transcripts in each group

Transcripts		T_2h vs. C_2h	T_6h vs. C_6h
**lncRNA**	up-regulated	35	31
	down-regulated	97	33
**mRNA**	up-regulated	878	755
	down-regulated	1177	972

### GO and KEGG analysis of lncRNA target genes

To explore the function of the identified lncRNAs, their potential target genes were predicted using *cis* (co-location) and *trans* (co-expression) methods. A total of 6493 *cis*-regulatory lncRNA-mRNA pairs were predicted within a region 100 kb upstream and downstream of lncRNAs, of which 1032 were within 10 kb upstream and downstream of the nearby target genes. GO analysis [44] showed that four GO terms were significantly enriched (corrected *p*-value < 0.05) in the T_2h vs. C_2h group. However, there was no significant enrichment of GO terms in the T_6h vs. C_6h group (Additional file 5: Table S5). KEGG analysis [45] indicated that 94 pathways were enriched in *cis*-regulatory target genes of lncRNAs in the two groups. The most enriched pathways included “Phosphatidylinositol signaling system,” “Ribosome biogenesis,” and “Glyoxylate and dicarboxylate metabolism.” The pathways “Hippo signaling pathway – fly,” “Notch signaling pathway,” and “Wnt signaling pathway” were common among the top 20 enriched pathways (Additional file 6: Table S6).

With respect to the *trans* regulation of lncRNAs, 42,797 *trans*-acting lncRNA-mRNA pairs were predicted, including 42,651 positive correlation lncRNA-mRNA pairs (*r* > 0.95) and 146 negative correlation pairs (*r* < − 0.95). There were 45 significantly enriched GO terms (corrected *p*-value < 0.05) in the T_2h vs. C_2h group, and none in the T_6h vs. C_6h group. Twenty GO terms associated with metabolism or biosynthesis were enriched in the category Biological Process, two in Cell Component and two in Molecular Function (Additional file 7: Table S7). KEGG analysis showed 98 pathways enriched in *trans*-acting target genes of lncRNAs in the two groups. The most enriched pathways were “Fatty acid biosynthesis,” “Citrate cycle,” and “Proteasome.” In the two groups, “Fatty acid biosynthesis,” “Fatty acid metabolism,” and “Citrate cycle” were the most enriched pathways among the downregulated target genes, while “Proteasome” and “Lysosome” were the most enriched pathways in the upregulated target genes. “Wnt signaling pathway” was the most common pathway among the top 20 enriched pathways in the T_6h vs. C_6h group (Additional file 8: Table S8).

### Functional analysis of mRNA in silkworm fat body

Differentially expressed mRNAs were analyzed using GO and KEGG enrichment. Seven GO terms were significantly enriched in the Biological Process and Molecular Function categories in the T_2h vs. C_2h group. Sixteen GO terms were significantly enriched in the T_6h vs. C_6h group (corrected *p*-value < 0.05), including 9 in Biological Process, 3 in Cell Component, associated with proteasome complex, and 5 in Molecular Function, related to different enzyme activities (Additional file 9: Table S9). KEGG analysis showed 113 pathways enriched in the differentially expressed mRNAs in the two groups. The most enriched pathways of mRNAs in the two groups were “Citrate cycle,” “Fatty acid metabolism,” and “Proteasome.” “Fatty acid metabolism,” “Fatty acid biosynthesis,” and “Citrate cycle” were the most enriched pathways for downregulated genes, and “Proteasome,” “Lysosome,” and “Peroxisome” were the most enriched pathways for upregulated genes. “Regulation of autophagy,” “Hippo signaling pathway–fly,” “Notch signaling pathway,” and “Jak-STAT signaling pathway” were the most common pathways in both groups (Additional file 10: Table S10).

### Validation and detection of differentially expressed lncRNAs and mRNAs

**Fig. 7:**
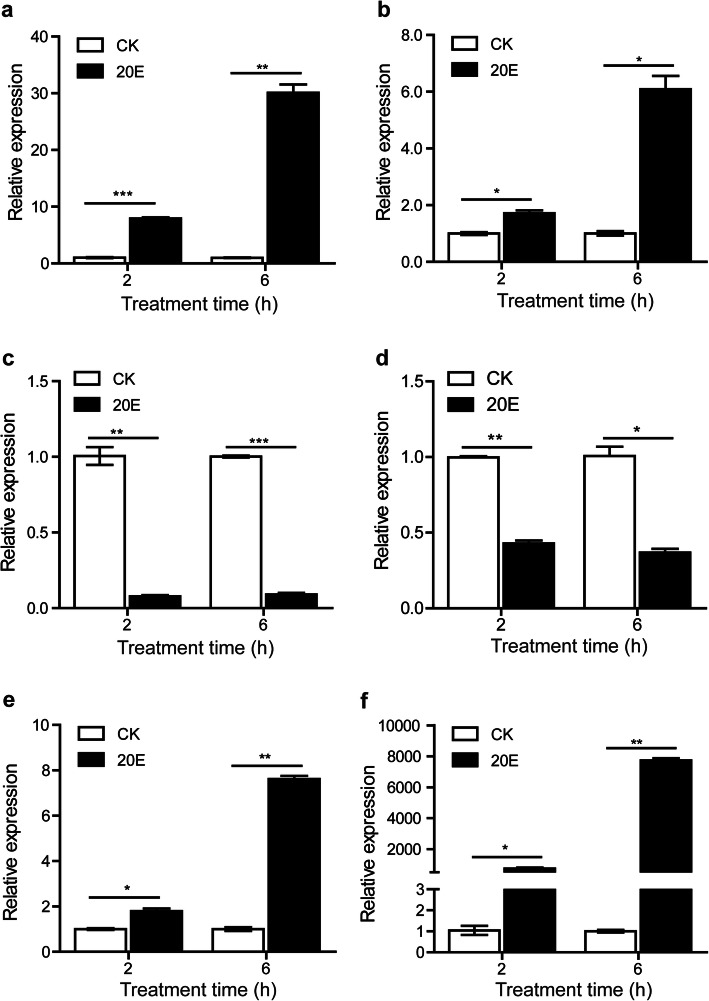
qRT-PCR validation of selected lncRNAs and mRNAs in 20E treated fat body. **a** *LNC_000560*, **b** *LNC_000063*, **c** *LNC_000458*, **d** *LNC_000585*, **e** *Atg4B*, **f** *HR3*. Data were normalized to the housekeeping gene *actinA3* and are shown as the mean ± standard error, *P < 0.05, **P < 0.01, ***P < 0.001, Two tailed, paired t test

To validate the RNA-seq results, four lncRNAs and two mRNAs were chosen for qRT-PCR analysis. *LNC_000560* and *LNC_000063* were significantly upregulated at 2 and 6 h.p.i.20E compared with control, whereas *LNC_000458* and *LNC_000585* were significantly downregulated. The predicted *trans* target genes (r > 0.95) of *LNC_000560* and *LNC_000063* were *Atg4B* and *HR3* respectively, which are essential proteins playing important roles in 20E regulated autophagy and the molting or metamorphosis of insects. As shown in Fig. 7, *LNC_000560*, *LNC_000063*, *LNC_000458*, *LNC_000585*, *Atg4B*, and *HR3* showed significantly different expression at 2 and 6 h.p.i.20E, and observation was consistent with the RNA-seq data.

**Fig. 8:**
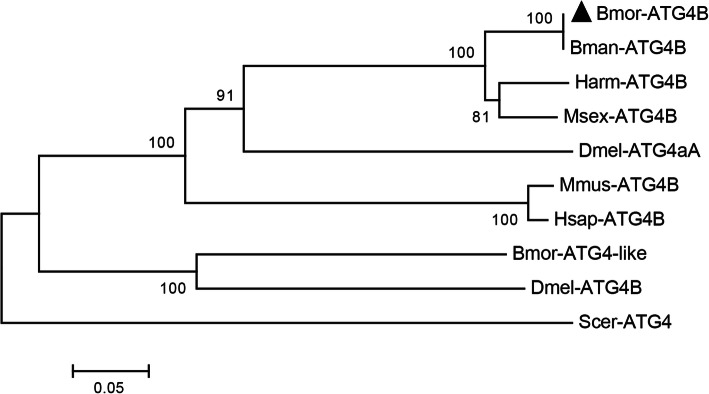
Phylogenetic analysis of the ATG4 homologs from different species. Bmor: *Bombyx mori* (Atg4B: XP_004929228.2; Atg4-like: ACJ46060.1), Bman: *Bombyx mandarina* (XP_028029080.1), Harm: *Helicoverpa armigera* (XP_021182852.1), Msex: *Manduca sexta* (XP_030033081.1), Dmel: *Drosophila melanogaster* (Atg4A: NP_608563.1; Atg4B: NP_650452.1), Mmus: *Mus musculus* (NP_777363.1), Hsap: *Homo sapiens* (AAH00719.1), Scer: *Saccharomyces cerevisiae* (NP_014176.2).

**Fig. 9:**
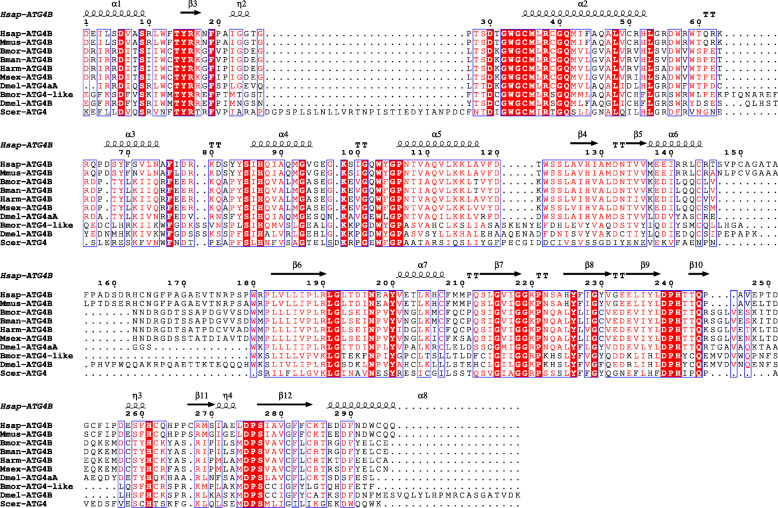
Alignment of peptidase C54 domain of ATG4 homologs in *Bombyx_mori* (Atg4B: XP_004929228.2; Atg4-like: ACJ46060.1), *Bombyx mandarina* (XP_028029080.1), *Helicoverpa_armigera* (XP_021182852.1), *Manduca sexta* (XP_030033081.1), *Drosophila_melanogaster* (Atg4A: NP_608563.1; Atg4B: NP_650452.1), *Homo sapiens* (PDB: 2D1I_A), *Mus_musculus* (NP_777363.1), and *Saccharomyces_cerevisiae* (NP_014176.2)

Because Atg4B has not been functionally identified in *B. mori*, the phylogenic analysis of ATG4B homologs from different species was performed. The results showed that Atg4B of *B. mori* was more conserved with the homologs from other lepidopteran species and *D. melanogaster* Atg4A. The sequence identitiy was 100 % identical to the Atg4B of *B. mandarina*, 88 % to *Helicoverpa armigera* and *Manduca sexta* Atg4B, 50 % to the *D. melanogaster* Atg4A, 47 % to *Homo sapiens* ATG4B, and 46 % to *Mus musculus* ATG4B, but only 33 % identities to the *B. mori* Atg4-like, 30 % to the *Drosophila* Atg4B, and 25 % to the *Saccharomyces cerevisiae* Atg4 (Fig. 8). The alignment of the sequences showed that the predicted functional domain, peptidase C54, was evolutionary conservation among the different ATG4 homologs (Fig. 9).

**Fig. 10:**
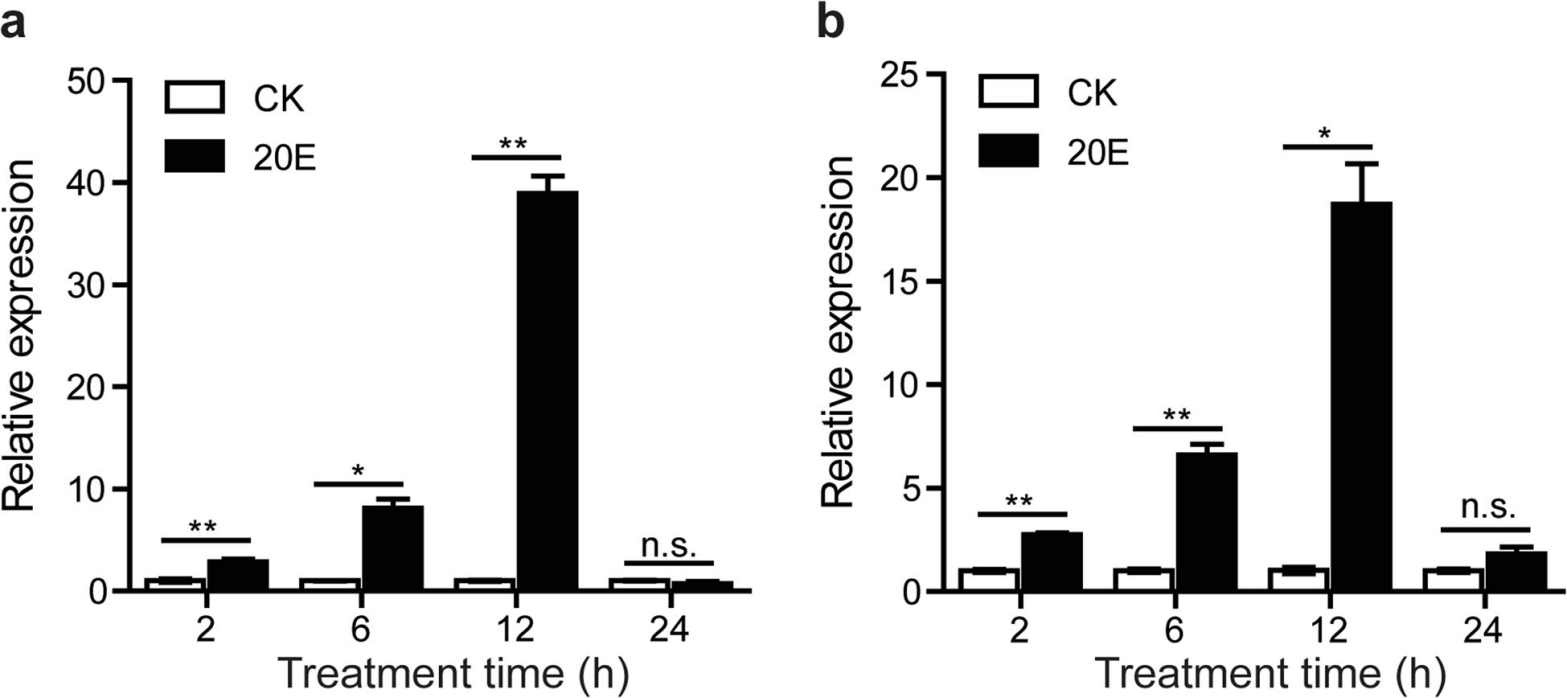
Expression profile of *LNC_000560* (**a**) and *Atg4B* (**b**) in 20E treated fat body of *B. mori* by qRT-PCR. Data were normalized to the housekeeping gene *actinA3* and are shown as the mean ± standard error, *P < 0.05, **P < 0.01, no significant differences are denoted by n.s. above bars, Two tailed, paired t test

Since Atg4B is an essential protein involved in autophagy, to further study the function of lncRNA in 20E-induced autophagy, we focused on *LNC_000560* and its target gene *Atg4B* to explore the regulatory function of lncRNAs in the silkworm. We further analyzed the expression profiles of *LNC_000560* and *Atg4B* in the fat body at 2, 6, 12, and 24 h.p.i.20E, at different developmental stages and in different tissues of silkworm larvae. *LNC_000560* and *Atg4B* showed highly similar expression patterns in the fat body. The expression of *LNC_000560* and *Atg4B* was significantly increased at 2, 6, and 12 h.p.i.20E, and decreased to basal levels at 24 h.p.i.20E (Fig. 10).

**Fig. 11:**
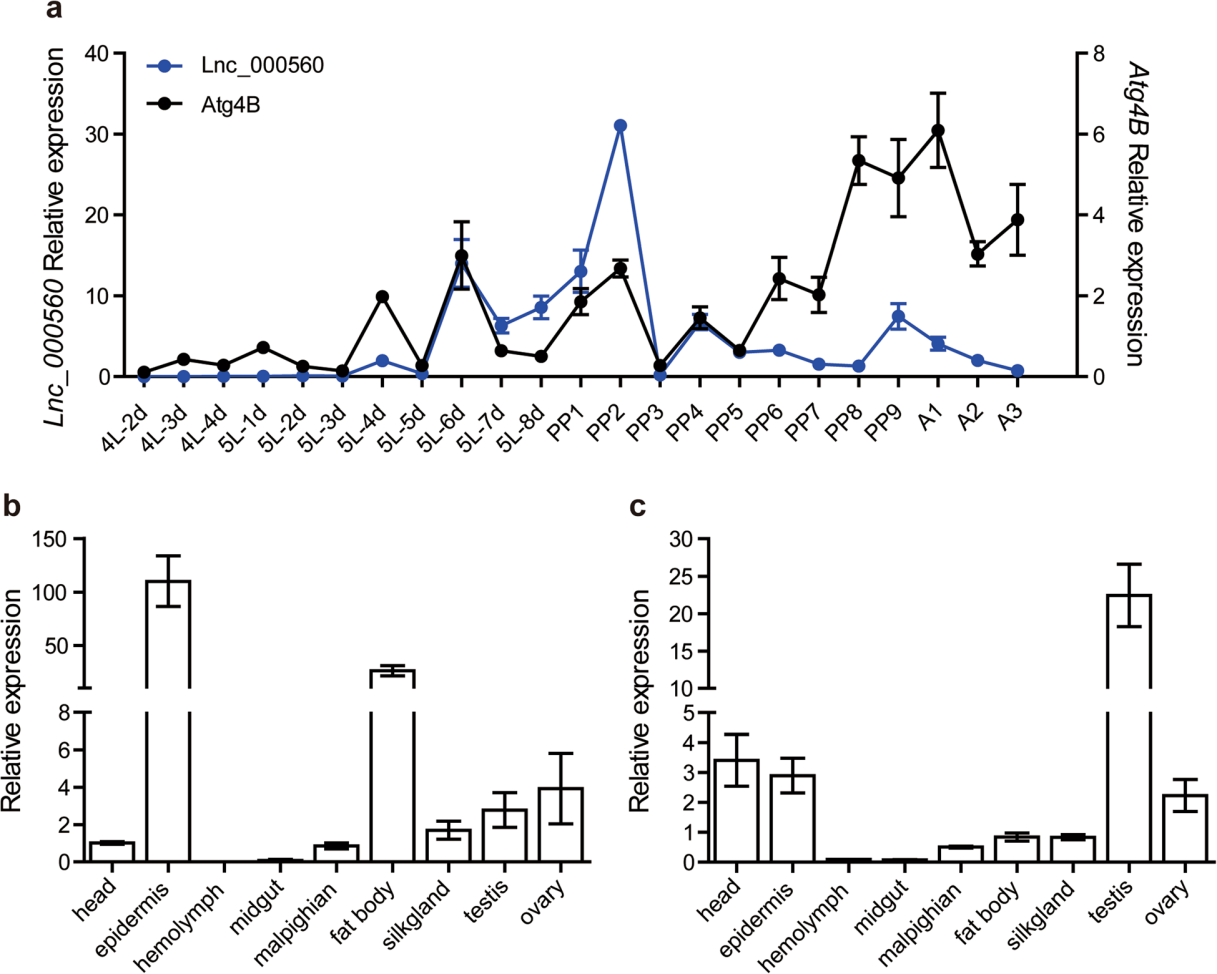
Expression profile of *LNC_000560* and *Atg4B* at different developmental stages (**a**) and in different tissues of 5th instar larvae (**b, c**) of *B. mori* by qRT-PCR. 4 L-2d to 5 L-8d represent day 2 of the 4th instar larvae to day 8 of the 5th instar larvae respectively; PP1-PP9 represent day 1 to day 9 of the pupal stages respectively; A1-A3 represent day 1 to day 3 of the adults respectively. Data were normalized to the housekeeping gene *actinA3* and are shown as the mean ± standard error

Their expression trend at different developmental stages was also consistent, while several peaks were detected from the second day of 5th instar larvae to the adult stage. Especially both exhibited expression peaks at the later stage of larvae and pupae, as well as on the second day of the pupal stage (Fig. 11a). The expression of *LNC_000560* in larval tissues was high in the epidermis and fat body, and relatively low in other tissues, with no expression in the hemolymph or midgut (Fig. 11b). *Atg4B* showed a similar expression pattern as *LNC_000560*, although its highest expression was observed in testis (Fig. 11c).

### RNA interference of lncRNA

**Fig. 12:**
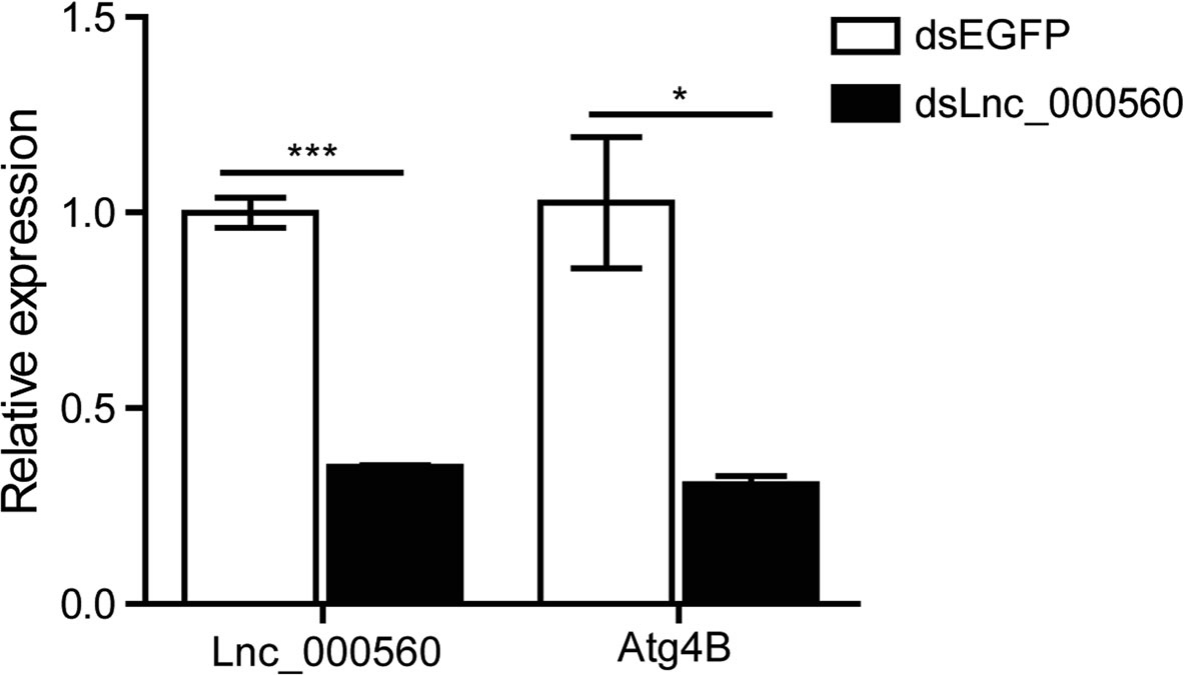
Expression of *LNC_000560* and *Atg4B* after RNAi of *LNC_000560* in fat body of 5th instar larvae of *B. mori* by qRT-PCR

To further study the regulation relation between *LNC_000560* and *Atg4B*, we performed RNAi experiment to knockdown the expression of *LNC_000560* in the fat body of silkworm larvae. Compared with dsEGFP control, we found that the expression of *LNC_000560 and Atg4B* in fat body was significantly decreased after LNC_000560 knockdown, which indicated that the expression of *LNC_000560* was successfully down-regulated and it could indirectly participate in the regulation of autophagy by regulating its target gene *Atg4B* (Fig. 12).

## Discussion

In insects, the fat body undergoes dramatic changes, via a process known as fat body remodeling, during metamorphosis from larva to pupa [34, 46, 47]. Fat body remodeling is regulated mainly by 20E and the AMPK-TOR pathway [48]. To identify lncRNAs involved in 20E-induced autophagy in the fat body, we chose the 2 and 6 h.p.i.20E experimental times for RNA-seq analysis. A total of 1035 putative lncRNAs were obtained, including 905 lincRNAs and 130 antisense lncRNAs. Previous studies have identified 11,810 lncRNAs from 21 different tissues [36], 6281 lncRNAs from the available RNA-seq data of *B. mori* [37], 599 lncRNAs in the silk gland [38], 13,159 lncRNAs in BmN cells [41]. The divergence of lncRNAs numbers in *B. mori* may be due to the use of different samples and methods. Most of the lncRNAs were located in intergenic regions, and had 2–3 exons, which were consistent with 65.6 % lincRNA in BmN cell and 74.83 % in silk gland. LncRNAs, especially antisense lncRNA, were longer than mRNAs, although they possessed a lower number of exons, which were also similar to those observed in previous studies [36–38]. Silkworm lncRNAs showed lower expression levels than protein-coding mRNAs observed, as in mammals [49].

Differential expression analysis showed that 166 differentially expressed lncRNAs and 3041 mRNAs appeared to be related to the 20E response, among which 30 differentially expressed lncRNAs and 741 such mRNAs were shared between the two groups (T_2h vs. C_2h and T_6h vs. C_6h). The *cis* and *trans* method was used to predicted the function of the identified lncRNAs, we found 6493 lncRNA-mRNA pairs in the *cis* prediction and 42,797 lncRNA-mRNA pairs in the *trans* prediction. KEGG analysis showed that most of the pathways enriched in *cis*-regulatory genes were different from those enriched in *trans*-acting genes. The pathways most enriched in differentially expressed mRNAs were “Citrate cycle,” “Fatty acid metabolism,” and “Proteasome,” similar to the *trans*-acting target genes. Therefore, we speculated that the identified lncRNAs might function mainly as *trans*-acting genes rather than as *cis*-regulatory genes. Besides, among 42,797 *trans*-acting lncRNA-mRNA pairs, more than 99 % of them were positive correlation. The pathways most enriched in both downregulated *trans*-acting target genes and mRNAs were “Fatty acid biosynthesis,” “Fatty acid metabolism,” and “Citrate cycle,” whereas, those enriched in upregulated genes were “Proteasome” and “Lysosome.” The insect fat body is the main organ involved in energy metabolism and is analogous to the adipose tissue and liver in vertebrates. In *Drosophila*, the fat body is a crucial tissue controlling energy storage and utilization, and meeting the changing energy demands of all developmental stages. Lipids, composed of fatty acids and cholesterol, are the main form of energy storage [47]. In the last decade, several studies have found that the expression of many genes involved in energy metabolism is downregulated by 20E in the *Drosophila* midgut and fat body, blocking their metabolic activity for the initiation of metamorphosis [50, 51]. Further evidence in silkworm indicated that the fat body is a responsive tissue, which modifies metabolic activity in response to molting and pupation induced by 20E [52]. Autophagy-lysosome and ubiquitin-proteasome are the two major protein degradation systems in all eukaryotic cells. They are responsible for maintaining protein homeostasis by degrading intracellular proteins [53]. Hence, we assume that the identified lncRNAs may function mainly through the positive correlation target genes, and the downregulation of these genes may play a role in fat body development, and upregulated genes may function mainly in the degradation of intracellular proteins via autophagy or ubiquitin pathway.

Several lncRNAs have been identified in the silkworm, but only a few of them have so far been functionally characterized [35, 39, 40]. We predicted 71 *trans*-regulated target genes for *LNC_000560*, 8 for *LNC_000063*, 12 for *LNC_000458*, and 50 for *LNC_000585*. We selected *LNC_000560* and *LNC_000063* to verify the expression of their target genes in the fat body. The results showed that *Atg4B* and *HR3* were significantly up-regulated, similar to *LNC_000560* and *LNC_000063*.

Atg4B, a target gene of *LNC_000560*, is the main human ortholog of four different ATG4 family members involved in autophagy and can cleave most of the human ATG8 homologs [54]. Increases in ATG4B levels are accompanied by the induction of autophagy in pneumonia and fibrosis [55]. The phylogenic analysis and functional domain alignment indicated that, apart from the Atg4B of lepidopteran species, the *B. mori* Atg4B was more conserved with *D. melanogaster* Atg4A and mammals ATG4B, and *B. mori* Atg4B-like was more conserved with *D. melanogaster* Atg4B. The functional difference between Atg4B and Atg4B-like in *B. mori* requires more investigation. In *B. mori*, a previous study demonstrated that *Atg4-like* is the least sensitive to 20E of the 13 *Atg* genes [10]. In our study, *Atg4B* was significantly up-regulated after 20E treatment, which indicated that *Atg4B* expression was more sensitive to 20E than *Atg4-like*. The expression profiles of *LNC_000560* and *Atg4B* were highly consistent in 20E-treated fat bodies and in the different developmental stages from larvae to adults. Moreover, RNAi of *LNC_000560* significantly decreased the mRNA level of *Atg4B*. While autophagic activity is reduced in *Atg4* deficient yeast and *Atg4b* deficient mice, the deletion of *Atg4c* in mice has little impact on autophagy, *Atg4d* silencing prevents autophagosome formation and induces cell death [54–56]. Therefore, *LNC_000560* may be involved in the 20E-induced autophagy of the fat body by up-regulating its target gene *Atg4B*. However, it remains unclear how *LNC_000560* regulate the expression of its target genes and participate in autophagy and the development of *B.mori.* Further studies should be conducted to clarify the regulatory mechanism between lncRNAs and their target gene.

## Conclusions

In the current study, 1035 lncRNAs were identified from 12 RNA-seq libraries of the *B. mori* fat body, including 905 lincRNAs and 130 antisense lncRNAs. In total, 166 lncRNAs showed significant changes in expression when responding to 20E. Prediction of target genes showed that some lncRNAs and mRNAs were involved in important biological processes, such as autophagy and molting. The functional study of *LNC_000560* and its target genes indicated a potential role of *LNC_000560* in 20E-induced autophagy of *B. mori* via the indirect regulation of Atg4B. These results provide a solid foundation for further study into the regulatory mechanisms of lncRNAs in 20E-induced autophagy, and their roles in other biological processes in *B. mori*.

## Methods

### Silkworm rearing and tissue collection

All experiments were carried out with silkworm strain Dazao p50, obtained from the Sericultural Research Institute, Chinese Academy of Agricultural Sciences. Larvae were reared on fresh mulberry leaves at 25℃ under 12 h light/12 h dark cycles.

The 20E levels in the 2-day-old 5th instar larvae are low, and the fat body is sensitive to 20E [57]. Accordingly, insects at this stage of development were chosen for injection of 5 µg 20E (Solarbio, SE8010) per larva. Control insects were injected with the same volume of solvent. The fat body tissues of ten individuals were dissected from the larval abdominal segment at 2, 6, 12, and 24 h.p.i.20E, and tissues from each time point were pooled. The insects’ whole bodies from each day of the 4th and 5th instar stages after discarding the food residues, as well as each day of pupae until the 3rd day of adulthood were collected. The head, epidermis, hemolymph, midgut, malpighian tubules, fat body, silk gland, testis, and ovary were collected from the 3rd day of 5th instar larvae. Three biological replicates were used for each experiment. All samples were frozen immediately in liquid nitrogen and stored at − 80℃ until use.

### LysoTracker Red staining

LysoTracker is an acidotropic dye that stains cellular acidic compartments, including lysosomes and autolysosomes. It has been used to detect autophagy-associated lysosomal activity from yeast to human [58, 59]. LysoTracker Red staining has proven to be an effective indicator of autophagy in *B. mori* fat body [10]. Newly collected fat body tissues were fragmented by forceps, washed with PBS, stained with LysoTracker Red DND-99 at a final concentration of 50 nM (L7528, Thermo Fisher Scientific, USA) for 5 min at 37℃, and washed again with PBS 3 times. The LysoTracker Red staining of the samples was observed under a Nikon Eclipse C1 confocal microscope.

### RNA extraction, library construction and sequencing

Total RNA from the silkworm’s fat body tissue at 2 and 6 h.p.i.20E was extracted using the Trizol reagent (Invitrogen) and further purified with RNeasy kits (Qiagen). RNA quantification and quantification were detected in Novogene Bioinformatics Institute (Beijing, China) following standard procedure. RNA purity was checked using a NanoPhotometer spectrophotometer (IMPLEN, CA, USA). RNA concentration was measured using Qubit RNA Assay Kits in a Qubit 2.0 Flurometer (Life Technologies, CA, USA). RNA integrity was assessed using RNA Nano6000 Assay Kits from the Bioanalyzer 2100 system (Agilent Technologies, CA, USA).

RNA-seq was carried out following standard Illumina protocols as previous reported [60]. A total of 3 µg RNA per sample was used as input material for RNA sample preparation. Ribosomal RNA was removed using Epicentre Ribo-zero™ rRNA Removal Kits (Epicentre, USA), and rRNA-free residue was cleaned up by ethanol precipitation. Subsequently, sequencing libraries were generated using the rRNA-depleted RNA with NEBNext Ultra™ Directional RNA Library Prep Kits for Illumina (NEB, USA), following manufacturer’s recommendations. Finally, the products were purified, and the library quality assessed using the Agilent Bioanalyzer 2100 system. The clustering of the index-coded samples was performed on a cBot Cluster Generation System using TruSeq PE Cluster Kits v3-cBot-HS (Illumina), according to the manufacturer’s instructions. After cluster generation, the libraries were sequenced on an Illumina HiSeq 2500 platform (Novogene Bioinformatics Institute, Beijing, China) and 125 bp paired-end reads were generated.

### Mapping to reference genome

Data analysis was conducted by the Novogene Gene Regulation Department (Beijing, China) as previous reported [60]. Raw read data in FASTQ format were processed using custom Perl scripts. In this step, clean data were obtained by removing reads containing adapter or ploy-N, and low-quality reads, from the raw data. The Q20, Q30, and GC contents of the clean reads were calculated. All downstream analysis were based on the clean, high quality reads. Reference genome and gene annotation files of silkworm *B. mori* were downloaded from SilkDB website (http://silkworm.genomics.org.cn/) and NCBI genome site (https://www.ncbi.nlm.nih.gov/genome/). An index of the reference genome was built using Bowtie v2.0.6 and paired-end clean reads were aligned to the reference genome using TopHat v2.0.9 (http://ccb.jhu.edu/software/tophat/index.shtml) [61].

### Transcriptome assembly

The mapped reads of each sample were assembled using both Scripture (beta2) [62] and Cufflinks v2.1.1 (http://cole-trapnell-lab.github.io/cufflinks/) [63] in a reference-based approach. Both methods use spliced reads to determine exon connectivity, but the methods use two different approaches. Scripture uses a statistical segmentation model to distinguish expressed loci from experimental noise, and uses spliced reads to assemble expressed segments. It reports all statistically significantly expressed isoforms at each locus. Cufflinks uses a probabilistic model to simultaneously assemble and quantify the expression level of a minimal set of isoforms which provides a maximum likelihood explanation of the expression data in each locus.

### LncRNA identification and classification

The putative lncRNAs were screened and identified from the silkworm transcriptome. The filtering process included five steps: (1) single exon transcripts were eliminated; (2) transcripts of ≤ 200 bp were removed; (3) transcripts that overlapped with any protein-coding exon in the sense orientation were filtered out; (4) transcripts with fragments per kilobase of transcripts per million mapped reads (FPKM) < 0.5 were removed; and (5) transcripts with protein-coding potential as predicted by either CPC or PFAM were excluded, while transcripts without protein-coding potential made up the candidate set of lncRNAs [64, 65]. The putative lncRNAs were classified into two groups, lincRNA and antisense lncRNA, using the class code module in Cuffcompare (http://cole-trapnell-lab.github.io/cufflinks/cuffcompare/) [66].

### Expression analysis of lncRNAs and mRNAs

The expression levels of both lncRNAs and protein-coding genes in each sample were measured using Cuffdiff (v2.1.1) [66]. Gene FPKMs were calculated by summing the FPKMs of transcripts in each gene group. FPKM stands for fragments per kilobase of exon per million mapped fragments, which is calculated based on the length of the fragments and reads count mapped to each fragment. Cuffdiff provides statistical routines for determining differential expression in digital transcript or gene expression data, using a model based on the negative binomial distribution. Transcripts with a *q*-value < 0.05 were identified as differentially expressed.

### Co-location analysis and functional enrichment

*Cis*-regulatory lncRNAs target and regulate the neighboring protein-coding genes [67, 68]. We searched the protein-coding genes within 100 kb / 10 kb upstream or downstream of all the identified lncRNAs, and then analyzed their function by functional enrichment. All co-located genes were used for GO enrichment analysis and KEGG enrichment analysis. GO enrichment analysis of the neighbor genes were implemented using goseq [69]. KEGG metabolic pathway enrichment analysis was carried out using KOBAS2.0 [70]. GO terms and KEGG pathways with *p*-values < 0.05 were considered significantly enriched.

### Co-expression analysis

The expression levels of the identified lncRNAs and mRNAs from the two different stages were used to analyze the co-expression relationships of lncRNAs and protein-coding genes. Pearson’s correlation coefficients between the expression levels of 1035 lncRNAs and 14,622 mRNAs were calculated with custom scripts (r > 0.95 or r < − 0.95), and the candidate lncRNA target genes were used for functional enrichment analysis to predict the function of lncRNAs. Hypergeometric test and Benjamini–Hochberg FDR (false discovery rate) correction were used for statistically analysis, and only GO terms or KEGG pathways with corrected *p*-values < 0.05 or FDR < 0.05 were considered significant.

### Expression validation of the selected lncRNAs and mRNAs

Atg1 is required for autophagosome initiation and is sensitive to 20E injection, Atg8 is the protein most widely used to monitor autophagy [71, 72]. The expression of the autophagy-related genes, *Atg1* and *Atg8*, in the fat body at 2, 6, 12, and 24 h.p.i.20E was evaluated using qRT-PCR. The relative expression levels of the differentially expressed lncRNAs was confirmed using qRT-PCR. Total RNA from silkworm fat body 2, 6, 12, and 24 h.p.i.20E, and different developmental stages and tissues were used for the first-strand cDNA synthesis using RevertAid First Strand cDNA Synthesis Kits (Thermo Fisher, USA), according to the manufacturer’s instructions. qRT-PCR was carried out using Roche FastStart Universal SYBR Green Master (Rox) (Roche, Switzerland). Each reaction was performed on a BIO-RAD CFX96 Real-Time PCR Detection System with three biological replicates. The relative expression levels of lncRNAs were calculated using the 2^−ΔΔCt^ method [73]. Silkworm *actinA3* (GenBank accession number U49854) was used as a reference gene. All the primers were designed using Primer 5.0 software (Additional file 11: Table S11). The PCR products were sequenced by the Beijing Genomics Institute (BGI, Beijing).

### RNA interference

The primers of *LNC_000560 and EGFP* containing the T7 promoter sequence were designed for RNA interference. Their double-stranded RNA (dsRNA) were synthesized using T7 RiboMAX™ Express RNAi system (Promega, P1700) according to the manufacturer’s instruction. The dsRNA (10 µg per larva) of *LNC_000560* was injected into 2-day-old 5th instar larvae, and *EGFP* dsRNA was injected as a control. After injection for 24 h, the fat body was dissected for qRT-PCR analysis. All the primers used in this study are listed in Additional file 11 (Table S11).

### Bioinformatics analysis

Phylogenetic analysis was performed using MEGA5.0 software and the neighbor-joining method. The alignment of the peptidase C54 domain of ATG4B homologs was performed using the programs MultAlin [74] and ESPript [75]. All sequences were obtained from the NCBI and SilkDB databases.

### Statistics analysis

Statistical analysis were performed using Prism 5, figures were prepared using Prism 5 and Adobe Illustrator CS5. The experimental data were analyzed using two-tailed, paired *t*-tests, *p < 0.05, **p < 0.01, ***p < 0.001. The values are shown as mean ± standard deviation of three independent experiments.

## Supplementary Information

**Additional file 1.** Summary of RNA-seq data of 12 silkworm samples.

**Additional file 2.** Detailed information of lncRNAs identified in this study.

**Additional file 3.** LncRNA and mRNA features.

**Additional file 4.** Common differentially expressed lncRNAs and mRNAs in the two groups.

**Additional file 5.** The significantly enriched GO terms detected in the two groups (*cis*).

**Additional file 6.** The top 20 enriched KEGG pathways in the two groups (*cis*).

**Additional file 7.** The significantly enriched GO terms detected in the two groups (*trans*).

**Additional file 8.** The top 20 enriched KEGG pathways in the two groups (*trans*).

**Additional file 9.** The significantly enriched GO terms of differentially expressed mRNAs in the two groups.

**Additional file 10.** The top 20 enriched KEGG pathways of differentially expressed mRNAs in the two groups.

**Additional file 11.** The primers of the selected lncRNAs and mRNAs.

## Data Availability

The datasets generated during the current study are available in the SRA database of the NCBI system with accession number of PRJNA672230 (https://dataview.ncbi.nlm.nih.gov/object/PRJNA672230?reviewer=um7or07tegs52t8kec6smbtne5).

## Abbreviations

LncRNA: Long non-coding RNA
20E: 20-hydroxyecdysone
Atg: Autophagy related
JH: Juvenile hormone
EcR: Ecdysone receptor
HR3: Hormone receptor 3
PI3K: Phosphatidylinositol 3-kinase
TORC: Target of rapamycin complex
h.p.i.: Hours post-injection
FPKM: Fragments Per Kilobase of exon model per Million mapped fragments
CPC: Coding Potential Calculator
PFAM: Protein Families Database
ORF: Open Reading Frame
GO: Gene Ontology
KEGG: Kyoto Encyclopedia of Genes and Genomes
AMPK: Adenosine 5’-monophosphate (AMP)-activated protein kinase
